# Successful Treatment of Traumatic Tetanus

**Published:** 1856-03

**Authors:** J. McF. Gaston

**Affiliations:** Columbia, S. C.


*Successful Treatment of Traumatic Tetanus.* By J. McF. Gaston, M. D., of Columbia, S. C.

In the latter part of the night of November 19th, a call came to my office from Ansell Abbott, a white youth about fifteen years old, who has been accustomed to out-door labor in our city. I was unable to attend at that time, and Dr. Talley, having visited him for me, has kindly furnished the particulars in reference to the case at its outset, in the annexed note:

*Dear Doctor:* At your request, I subjoin a brief abstract of the case of A., whom I visited for you during your indisposition.

Early in the morning of November 20th, I was called to see the patient. I found him laboring under symptoms of strongly marked and well developed Tetanus.

Nearly all the muscles of the body were in a state of rigid contraction. Those of the back, however, being in the ascendant, the entire body was flexed posteriorly in the form of an arch. The jaws were firmly fixed, so as to render impossible the protrusion of the tongue to the smallest extent. The abdominal muscles were as hard and unyielding as a board, and as clearly resonant under percussion. The pulse was small, frequent, and at times intermitting—in number one hundred and fifty pulsations to the minute. The bowels were constipated; the skin suffused with a cold, clammy perspiration. He complained of constant and acute pain in the region of the diaphragm, which was greatly agitated at every attempt at motion; as, indeed, was also the spasm of the muscles.

The history given me of his case was, that some time before (near three weeks) he had trodden upon a nail, which had entered the foot; but until within the last few days, he had experienced no inconvenience from it whatever; since which time, he began to perceive a stiffness about the neck and jaws, which had continued to increase until he found himself in the condition in which I then saw him.

I prescribed 60 drops of tincture of opium, to be repeated every second hour until he should sleep.

Some hours afterwards I again visited the patient, and finding no abatement of his symptoms, with your concurrence, I invited Dr. Trezevant to see him in consultation with me. This he did; and we determined to test the virtues of the cannabis indica, by ordering it in teaspoonful doses every hour, until its specific effects should be observed. With his sanction, I made a free incision into the sole of the foot, and directed a warm poultice to be applied.

Late on the afternoon of the same day you saw him with us. He had then taken nearly an ounce of the saturated tincture of the cannabis indica, without its affecting, to any perceptible degree, the rigidity of the muscles; and without procuring for him any diminution of his sufferings. His pulse still continued quick and feeble; his jaws immovable; and his body arched backward to its utmost stretch.

Of the plan of treatment then adopted, and subsequently pursued, you do not, of course, desire to be informed, as the conduct of the case, from that time, devolved upon you as his regular medical attendant.

Allow me to say, in conclusion, that I have rarely seen a more unpromising case of Traumatic Tetanus than that which I have above attempted to describe. Yours, very truly,

ALEX. N. TALLEY.

November 20th.—On the afternoon of this day, I saw the patient in the condition above described by Dr. Talley, with a pulse beating one hundred and fifty per minute; and with such rigidity of the extensor muscles as to induce complete opisthotonos. In the susequent treatment of this case, I had the advice of Dr. Talley regularly, and occasionally, also, that of Dr. Trezevant; and I make this acknowledgment at the outset, as there was a concurrence in the measures resorted to in each stage of the treatment; and I will simply state what was done, and the effect upon our patient's condition.

Chloroform was administered, by inhalation, until complete anæsthesia ensued—which was within five minutes from the beginning—and in a short time—not exceeding ten minutes—the pulse was reduced in frequency to one hundred beats per minute. The breathing was regular, and without oppression, under the influence of this agent; and as the tone of the circulation was good, we concluded to keep up the effect by repeated inhalations during the evening and night, and accordingly instructed the attendants to administer it whenever there should be a return of consciousness, or a tendency to spasmodic action, which had entirely subsided, leaving the muscles relaxed and the limbs flexible. The patient was kept in the anæsthetic state for twelve hours, and without any return of rigidity until the use of the chloroform was suspended on the following morning.

November 21st, 9 o'clock, A. M.—The frequency of the pulse and the spasmodic rigidity had returned to a considerable extent; and there was some nausea succeeding the use of the chloroform. Upon examining the foot, the original entrance of the nail was found in an irritable condition, and also a point above, towards which it had been directed in passing through the tissues between the tendons of the third and fourth toes; and it was deemed advisable to bring the patient under the influence of chloroform with a view to make incisions into those parts. Anæsthesia was not so readily induced now as formerly; but after some time, complete unconsciousness ensued, and the spasm of the muscular system was entirely controled, and the pulse reduced below one hundred per minute.

Deep incisions were now made into the palmar and dorsal points of irritation, and extended to some distance from each, in the line of the tendons. A warm hop poultice was directed to envelop the entire foot, which was then to be placed over a warm vapor bath for some hours, with a view to soothe and relax the tissues of the part, and thus promote suppuration.

Observing the nausea which had supervened upon the subsidence of the effect of the chloroform, we concluded that it could not be persevered in advantageously, and it was determined to resort to the lobelia inflata. The compound tinc. of lobelia inflatia, Cayenne pepper, gum myrrh, and rad, valerian, known amongst the Botanic school as third preparation, presented an eligible formula; and it was administered, at first, in the dose of a teaspoonful, in ginger tea, every hour; and after three hours, in the dose of a tablespoonful every honr, until *f*℥5 were consumed; when the interval was lengthened to two hours, and continued.

November 22d.—The patient was relaxed to a very considerable extent, and the pulse was reduced to eighty per minute; but the stomach became irritable toward evening, and to secure the retention of the medi cine in the system it was introduced by enemata; three tablespoonsful of the third preparation being mixed with the same quantity of fluid starch, was administered every three hours. The use of the medicine, by the mouth, was now interrupted; and an effort was made to have nourishment taken inco the stomach; but there was very little inclination to take any thing but cold water, which was forbidden, except in very small quantity.

November 23d.—Finding that the frequency of the pulse had increased from the previous day, and that there was some more tendency to rigidity and spasm of the muscles, it was determined to use the pulverized seed of lobelia with the starch, as an enema, instead of the tincture, which combined stimulating articles with it, besides the alcobolie vehicle.

As the sedative effects of the lobelia were desired, it was given in portions of a tablespoonful, with three of fluid starch, every six hours, and it seemed, for a time, to have considerable control over the rigidity of the muscles and the pulse.

November 24th.—The effect of the injections were manifested by irritability of the stomach, and thus its soothing influence was greatly impaired. To obviate this, tinc. opii. was given, by the mouth, in teaspoonful doses, after each enema of the lobelia seed, but without any perceptible effect in lessening the disturbance of the stomach.

November 25th.—As the irritability of the stomach continues, and seems likely to thwart the end in view, sulph. morph., gr. i, is ordered after each enema, every six hours. This has the desired effect, and is persevered in, with some occasional interruption, for four days. At the same time, milk is used as diet, and is generally retained better than any thing else which he attempts to eat.

November 29th.—The spasmodic rigidity of the muscles is very much diminished; the pulse is reduced in force; the irritability of the stomach has again increased; the general strength of the patient is evidently much impaired; and considerable emaciation is perceptible. The diseased action seems to have yielded to a great extent; but with it, the vital force has given way, and the patient seems unable to bear the prostrating effect of the sedative article which has been used up to this time. The lobelia is accordingly discontinued, and the sulph. morph., gr.$\frac{1}{2}$, is given every six hours until the following morning.

November 30th.—The stomach has now become quiet, but the tendency to prostration is very manifest, and a decidedly stimulating course of treatment is agreed upon. A combination of sulp. quin., ʒi, carb. amm., ʒij, tinc. opii. camph., *f*℥ij, water, *f*℥vi, in the dose of a tablespoonful every two hours, is given throughout the day and night, and with good effect.

December 1st.—There has been no increase of rigidity from the stimulant, and French brandy is added in the afternoon, in doses of three tablespoonsful, with milk, every two hours, alternating with the former medicine.

December 2d.—There is an evident improvement in the pulse, and in the strength of the patient, without any increase of rigidity. He now begins to desire nourishment, and takes a soft boiled egg, with bread and coffee.

December 3d.—The time is lengthened to three hours between each article, making an hour and a half from one to the other; and he now takes nourishment freely. The muscles are beginning to regain their power; and although there is considerable stiffness, it may be overcome by voluntary power directed to the part for some time. If he is turned on his back, the curvature is perceptible for a short time; but, as he expresses it, the back settles down to the bed after a while. The pulse ranges now about one hundred to the minute; but is variable, and seems to change in frequency, in different positions of the body, without reference to the disturbance of motion in changing the posture. It is less frequent when he lies on his back than when he lies on his abdomen—as he has been very prone to do throughout the attack, and still manifests the same disposition.

December 5th.—He is free from all uneasiness, and has regained the use of the muscles of the back and legs to such an extent as to turn himself in any position, and to rise up and sit in bed. The bowels are now regularly open during the day; a result which, during the spasmodic rigidity, did not occur more than once in four or five days, notwithstanding the constant use of enemata.

December 7th.—He takes nourishment freely, and is convalescent.—*Charleston Medical Journal.*

